# Anomalous Optical Properties of KTN:Li Ferroelectric Supercrystals

**DOI:** 10.3390/nano13050899

**Published:** 2023-02-27

**Authors:** Ludovica Falsi, Salvatore Macis, Yehonatan Gelkop, Luca Tartara, Eleonora Bonaventura, Paola Di Pietro, Andrea Perucchi, Yehudit Garcia, Galina Perepelitsa, Eugenio DelRe, Aharon J. Agranat, Stefano Lupi

**Affiliations:** 1 Dipartimento di Fisica, Università di Roma “La Sapienza”, 00185 Rome, Italy; 2 The Department of Applied Physics, The Hebrew University of Jerusalem, Jerusalem 9190401, Israel; 3 Dipartimento di Ingegneria Industriale e dell’Informazione, Università di Pavia, 27100 Pavia, Italy; 4 CNR-IMM Unit of Agrate Brianza, 20864 Agrate Brianza, Italy; 5 Elettra—Sincrotrone Trieste S.C.p.A. S.S.14, Km 163.5 in AREA Science Park IT-34149 Basovizza, 34100 Trieste, Italy; 6 ISC-CNR, Università di Roma “La Sapienza”, 00185 Rome, Italy

**Keywords:** nanodisordered perovskite, spectroscopy, supercrystal, nonlinear optics, giant refraction

## Abstract

We report a spectroscopic investigation of potassium–lithium–tantalate–niobate (KTN:Li) across its room-temperature ferroelectric phase transition, when the sample manifests a supercrystal phase. Reflection and transmission results indicate an unexpected temperature-dependent enhancement of average index of refraction from 450 nm to 1100 nm, with no appreciable accompanying increase in absorption. Second-harmonic generation and phase-contrast imaging indicate that the enhancement is correlated to ferroelectric domains and highly localized at the supercrystal lattice sites. Implementing a two-component effective medium model, the response of each lattice site is found to be compatible with giant broadband refraction.

## 1. Introduction

Ferroelectric KTN:Li cooled to its room-temperature phase transition manifests a ferroelectric supercrystal (SC) [1,2,3,4,5,6,7,8], a complex orderly multidomain state with the potential to provide hereto unavailable functionalities in key applicative scenarios, such as for energy and information storage [9,10,11,12]. One model of the SC is that of a volume lattice of 3D spontaneous polarization vortices (see, for example, Ref. [1]). The structure at once obeys closed-flux conditions associated with volume charge compensation and elasto-mechanical equilibrium, and is compatible with X-ray diffraction and second-harmonic-generation (SHG) experiments [13,14]. For propagating beams, SCs manifest giant broadband refraction (GR), an intriguing and as yet unexplained phenomenon by which focused visible light, even incoherent white light, is observed to propagate without diffraction along the normal to the input of a zero-cut sample facet, irrespective of launch angle, wavelength, intensity, and size of the input beam [15]. While a large index of refraction is excluded in homogeneous substances [16], GR is observed to be a strongly inhomogeneous effect, occurring, in terms of the SC 3D model, in the vortex/anti-vortex (saddle-point) cores of the spontaneous polarization distribution.

No spectroscopic evidence of GR in a sample manifesting a SC has been reported. Indeed, anomalous optical response connected to the formation of ferroelectric domains should leave a trace in the sample’s spectroscopic scan. For one, the emergence of the ferroelectric phase with its birefringent polar clusters and domains will strongly affect both reflection spectroscopy and the infrared and Raman spectrum [17,18,19,20]. In KTN, this is exemplified by the strong correlation of Fano resonances in the Raman spectra with even minute changes in composition and doping [20,21,22].

We here report detailed results retrieved from a broadband Infrared (IR)–Ultraviolet (UV) spectroscopy study of KTN:Li across the room-temperature ferroelectric phase transition. An anomalous optical response is found in proximity of the Curie point, an enhancement of the real part of the index of refraction for a specific broad band that spans visible and near-infrared wavelengths, with negligible absorption. This band is found to coincide with that of SHG at the Curie point, the implication being that the enhancement is connected to the underlying polar domain distribution. Phase-contrast imaging of the underlying supercrystal indicates a periodic array of localized high index of refraction regions embedded in a standard index of refraction host. A two-component analysis of the spectroscopic data fixes the spectral band of enhancement to 700–1100 nm, centered at around 880 nm. The enhanced index component becomes compatible with GR results for a mixing ratio of 1% of the bulk crystal interface, a ratio that is in agreement with phase-imaging results.

## 2. Results

Broadband optical measurements of the KTN:Li sample (see Section 4.1) have been performed using three different experimental apparatus: a Bruker Vertex 70v interferometer from the mid infrared (MIR) to the near infrared (NIR) spectral region, a Jasco V770 spectrometer from NIR to the UV region (see Section 4.2). For each spectral region where both reflectance *R* and transmittance *T* are nonvanishing, the real (*n*) and imaginary (*k*) part of the refraction index versus the incident electromagnetic field wavelength λ is extracted from the reflectance *R* and transmittance *T* data.

**Figure 1:**
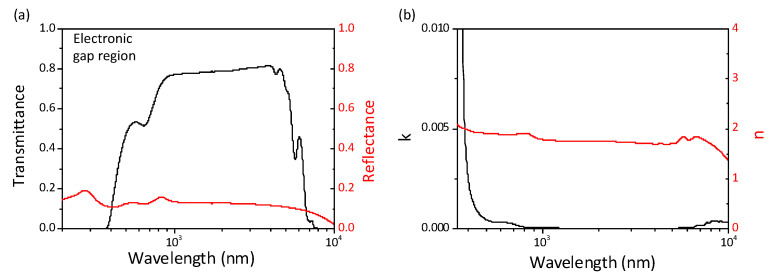
Broadband optical spectroscopy measurements from IR to UV of paraelectric KTN:Li ($T>T_{C}$). (**a**) Measured transmittance and reflectance suggests a dielectric behavior with a short wavelength electronic gap. (**b**) Corresponding values of real *n* and imaginary *k* index of refraction. Since in the electronic gap region the trasmittance vanishes, we extract *n* and *k* in the range 350–10${}^{4}$ nm.

Figure 1 reports broadband reflectance and transmittance measurements at room temperature, in the paraelectric phase (RT = 25 ${}^{\circ }$C > $T_{C}$). Results refer to a spatially averaged response over macroscopic regions of the sample (size of the beam used was smaller than the sample size ≃ 1.7 mm). Paraelectric KTN:Li behaves as a dielectric. In detail, the high frequency spectrum signals an electronic gap at around 400 nm (Figure 1a), while the visible spectrum is characterized by high transmittance, with a corresponding real and imaginary part of the refractive index n ∼ 2.3 and $k<10^{-4}$ (Figure 1b). Here, *n* and *k* are derived using the fully analytical solution to the inverse problem, a set of equations providing *n* and *k* values from *R* and *T* of a slab with parallel polished faces [23,24].

**Figure 2:**
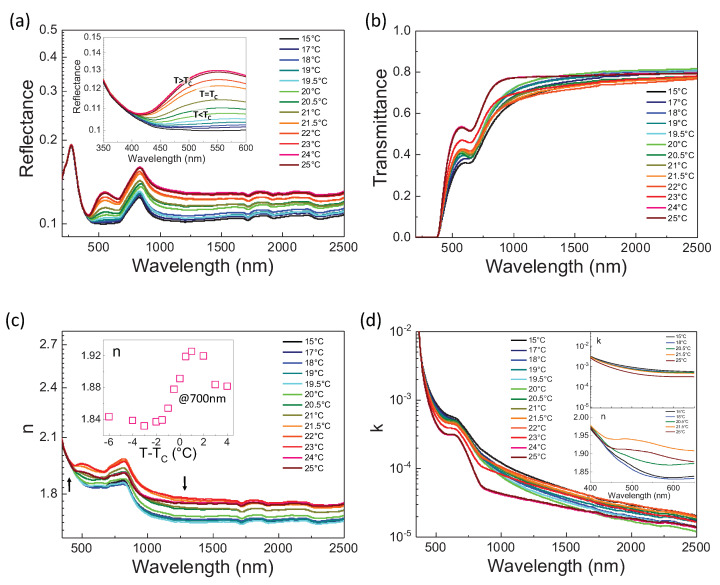
Optical spectroscopy of KTN:Li across the ferroelectric phase transition. UV−IR Reflectance (**a**) and Transmittance (**b**) in passing from $T>T_{C}$ to $T<T_{C}$. Corresponding real *n* (**c**) and imaginary *k* (**d**) index of refraction. Inset in panel (**c**) reports *n* versus $T-T_{C}$ for a specific wavelength (700 nm), while black arrows indicate, correspondingly, the beginning and the ending of the exotic temperature dependence. Note the peak in *n* at 21.5 ${}^{\circ }$C with no corresponding feature in *k*. The response in the visible (500–900 nm) in panels (**c**,**d**), while deduced analytically, seems qualitatively in contrast with common dielectric response [16].

The picture is fundamentally altered as the sample is cooled through the Curie point $T_{C}$. This is evident in the infrared-UV spectrum versus temperature reported in Figure 2 performed using the JASCO setup. The reflectance *R* (Figure 2a) and transmittance *T* (Figure 2b) manifest an exotic non-monotonic relative temperature dependence that translates into an equally exotic behavior for the real (Figure 2c, see inset for a sample T scan at 700 nm) and imaginary part (Figure 2d) of the average refractive index. Specifically, an enhancement in the real part of the *n* in the band 500nm and 1200nm at $T_{C}$ emerges that is accompanied by only a slight variation in the corresponding imaginary part, that does not manifest an anomaly and remains limited to low values (Figure 2d, see inset). Indeed, the low values of *k* in Figure 2d indicate that scattering, including possible coherent Bragg-like scattering associated with the lattice of ferroelectric domains, plays a negligible role. Furthermore, strong temperature dependence means that material and impurity absorption play a marginal role in the anomalous response (see inset in Figure 2c).

**Figure 3:**
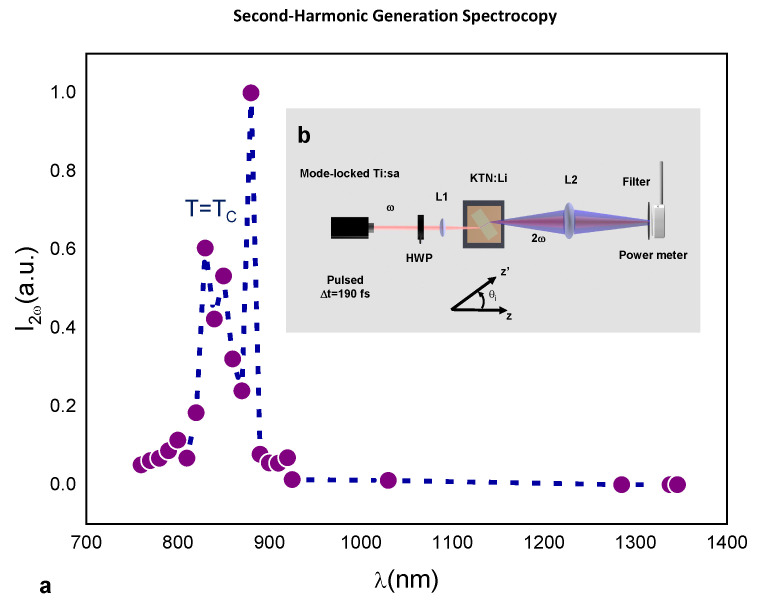
SHG Spectroscopy. (**a**) Converted signal power $P_{2\omega }$ versus λ. (**b**) Experimental setup (see Section 4.3).

Recent studies in bulk KTN show how ferroelectric domain distribution can considerably affect spectroscopic response in the visible and near-infrared spectrum [25]. To test the role of domains in the anomalous optical response, we measured SHG for a wide range of signal wavelengths [7,26,27,28,29]. SHG conversion versus wavelength is reported in Figure 3 (see Section 4.3). SHG power (full circles in Figure 3a) increases with λ reaching the maximum peak at λ = 880 nm. For longer wavelengths, the SHG power decreases, becoming negligible for λ>1285 nm. The SHG band in Figure 3a is contained within the band leading to anomalous response reported in Figure 2c.

**Figure 4:**
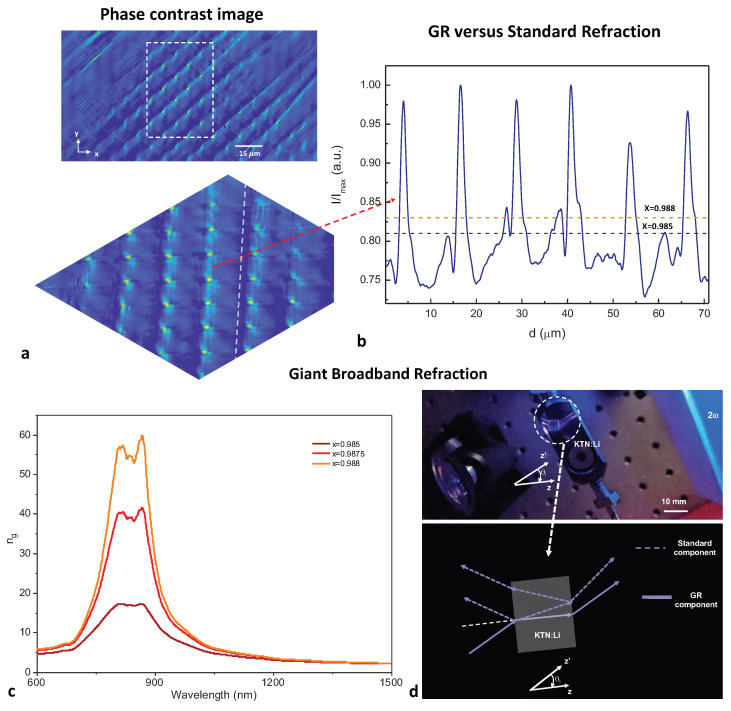
Giant broadband refraction in the SC lattice sites. (**a**) Top panel: Wide-area phase-contrast image of the KTN:Li sample showing the regions on the SC lattice sites that manifest strong index of refraction enhancement at $T_{C}$; bottom panel: enlarged rendering of selected region (dashed rectangle in top panel). (**b**) Intensity profile of the phase-contrast image along the dashed line of (**a**) (bottom panel). Two sample threshold levels are shown, with corresponding expected values of *x* (horizontal dashed lines). (**c**) Comparison of $n_{g}$ for different values of *x*, as derived from the analysis of the index of refraction. (**d**) Snapshot showing propagation along the normal to the crystal facet (i.e., along *z*) for a pump launched at a finite angle θ (along the $z^{\prime }$ axis) and illustration.

To analyze the spatial distribution of the anomalous index of refraction behavior, we used phase-contrast microscopy. This is achieved by means of an Olympus BX51 optical microscope implementing a two-dimensional double-sideband phase contrast technique (see the Section 4.4). The results reported in Figure 4a show that the high refraction index ($n_{g}$) regions, flagged in the phase-contrast image by lighter false colors, are localized on a lattice structure compatible with a self-organized SC (of lattice constant Λ≃ 15 µm) [1,15]. While the periodic structure is reminiscent of a photonic crystal lattice [30,31], an explanatory picture based on coherent Bragg-like resonances is incompatible with the broadband nature of the spectroscopic anomalies (Figure 2). In turn, the fact that the increased index of refraction emerges in localized regions of the sample suggest that optical response on the macroscopic (hundreds of micrometers) scale can be analyzed using a two-component model, i.e., a fraction *x* of the sample surface with a standard index of refraction $n_{0}$ and a fraction 1−x with an enhanced index $n_{g}$. The phase-contrast image itself can be used to provide an estimate of 1−x, as illustrated in Figure 4b, by considering a region to belong to the 1−x fraction if the associated phase-contrast intensity is above a threshold. Two different choices of threshold are shown in Figure 4b (horizontal dashed lines), resulting in two slightly different estimates of *x*.

In these terms, we expect the measured values of *n* to be the result of the effective medium model [32,33]

(1) $$x\frac{n_{0}-n}{n_{0}+2n}+\left(1-x\right)\frac{n_{g}-n}{n_{g}+2n}=0.$$

Assuming $n_{0}=n\;(25{\;}^{\circ }$C $>\;T_{C})$, one can evaluate $n_{g}$ in a broad spectral range from the relationship

(2) $$n_{g}=\frac{2n^{2}+\left(1-3x\right)n\cdot n_{0}}{n_{0}+\left(2-3x\right)n}.$$

Values of $n_{g}$ for different values of *x* are reported in Figure 4c (solid lines). In the case of x=0.988 (Figure 4b), a broadband giant index of refraction emerges from 450 to 1100 nm, with a plateau from 800 to 900 nm where $n_{g}>50$, a giant index of refraction that is compatible with the truly anomalous dynamics of propagating light beams, a sample of which is reported in Figure 4d. Specifically, the signal beam is observed to propagate along the normal to the input sample facet while SHG, the blue hue in the image of the scattered light, forms a wide-angle emission at output.

## 3. Discussion and Conclusions

We emphasize that while these findings provide indirect evidence of GR in KTN:Li, the physical origin of the effect remains unclear. Indeed, if GR can be understood and therefore harnessed, it can pave the way to a new class of applications based on large and accessible values of broadband index of refraction *n*. For one, with n≫1, a scaling of the visible effective wavelength λ/n down to tens of nanometers would allow the shrinking, in a self-similar Lilliputian-like transformation, of standard photonics to the nanoscales. The material could then host beams with a nanoscale cross-section, one very big step towards that ultimate goal of atom-mediated photon-to-photon coupling, the key to scalable optical quantum computers [34]. This is equally the case for imaging, where nanoscale details could be processed without resorting to super-resolution [35,36]. In metasurface optics, a small λ/n could allow ultra-rapid phase oscillations and wavefront reshaping over proportionally shorter distances [37,38,39,40,41]. In light harvesting, high-index ultrathin solar cells could outdo the so-called 4$n^{2}$ limit [42,43], while for pulses, a large *n* could support a strongly reduced group velocity, further enhancing light–matter interaction [44] and wavelength conversion [45,46].

In summary, we report an ultra-wide-band spectroscopic analysis of bulk crystal KTN:Li. At the Curie point, results indicate an anomalous behavior in the visible and near-infrared spectrum. Using phase contrast, SHG spectroscopy, and a two-component model, spectroscopic data are compatible with the emergence of a spatially inhomogeneous giant index of refraction experienced by light propagating in proximity of the supercrystal lattice sites. Arising in a not yet fully explored state of matter, that of ferroelectric supercrystals and superlattices, our result represents further evidence of anomalous response that sides other remarkable and unusual behaviour, such as negative capacitance [47] and enhanced ferroelectricity [9], with potential applications in the implementation of miniaturized electro-optic devices, and for charge storage and tunable capacitors [27].

## 4. Methods

### 4.1. Material

The sample we have grown is a zero-cut polished lithium-enriched solid solution of potassium–tantalate–niobate (KTN:Li) with an average composition K${}_{0.997}$Ta${}_{0.64}$Nb${}_{0.36}$O${}_{3}$:Li${}_{0.003}$. The zero-cut polished 2.50(a)×1.70(b)×2.03(c) mm sample has a cubic-to-tetragonal (m$\bar{3}$m to 4 mm) ferroelectric phase transition at $T_{C}=294$ K (21 ${}^{\circ }$C). The unit cell manifests random substitutions, a compositional disorder that, on consequence of the structural flexibility typical of perovskites, leads to locally modified polarizabilities and temperature-dependent nanoscale dipolar structures (nanodisordered ferroelectricity). The result is a modified ferroelectric behavior dominated by so-called polar nanoregions (PNRs), characterized by dielectric dispersion and out-of-equilibrium behavior (relaxor ferroelectricity) [48,49]. In our present case, this disorder is itself not homogeneous, manifesting a spatially periodic micrometric oscillation along a specific crystal axis. This is because the sample is grown into a bulk through the top-seeded method, a technique that entails a slight time oscillation in the temperature of the solidifying melt that, in turn, translates into an approximately periodic 7.5 µm striation grating along the growth axis (the *a* axis) [50,51]. This pattern conditions the nanoscale dipolar structures that, for the range $T_{C}>T>T_{C}-3$K, form a three-dimensional mesh of spontaneous polarization, the supercrystal [1].

### 4.2. Optical Setup

For the RT measurement, we used a Vertex 70v Bruker interferometer, covering the MIR-NIR region from $1000\;{\mathrm{cm}}^{-1}$ (10 µm) up to $3333\;{\mathrm{cm}}^{-1}$ (3 µm) with a resolution of $4\;{\mathrm{cm}}^{-1}$. The sample is kept in a low vacuum environment (∼$10^{-2}\;\mathrm{mbar}$ in order to eliminate the vibrational features of CO${}_{2}$ and water vapor). Both RT and temperature-dependent measurements at higher frequency were performed using a JASCO 760v spectrometer, covering the NIR-UV spectral region from 3000nm to 200nm. The whole reflectance and transmittance spectra (200 nm to 100 µm in wavelength) were obtained merging the different spectral ranges, which showed a very good superposition. The temperature control of the sample was managed via a water-based heat-exchange setup in the 15–25 ${}^{\circ }$C range. Measurements were performed in heating mode, increasing the temperature with a 0.2 ${}^{\circ }$C/min rate and dwelling 5 min at each temperature in order to stabilize the system, with an accuracy down to 0.1 ${}^{\circ }$C.

### 4.3. SHG Setup

SHG experiments were conducted in the 760–930 nm range using a Tsunami Spectra Physics Ti:Sa CW mode-locked laser (maximum output power of 0.6 W at λ=810±7 nm), with a repetition rate of 80 MHz and a pulsewidth of 190 fs (see scheme illustrated in Figure 3b). At 1030 nm, SHG was observed using a diode-pumped ultrafast Yb:KGW laser with a maximum output power of 0.6 W, a repetition rate of 108 MHz, and a pulsewidth of 150 fs. Measurements in the 1200–1400 nm range were performed using a synchronous pumping OPO (maximum output power of 0.3 W) with a repetition rate of 82 MHz and a transform-limited pulsewidth of 150 fs. Laser beam linear polarization, TM or TE, was set using a λ/2 waveplate (HWP), while the beam was focused onto the input facet of the $\theta_{0}$-rotated sample using a 50-millimeter-focal-length lens. The pump beam was focused to an input FWHM ≃15 µm, and the converted signal was measured filtering and focusing onto a power meter.

### 4.4. Phase-Contrast Imaging

White light passing through an annular phase plate was focused onto the sample surface and collected in transmission using an oil immersion 100× objective. The sample itself was placed onto a sapphire window and kept at a fixed *T* ≃ $T_{C}$.

## Data Availability

All the data supporting the results presented in this paper are available from the corresponding author upon reasonable request.
